# Simultaneous Detection of Fenitrothion and Chlorpyrifos-Methyl with a Photonic Suspension Array

**DOI:** 10.1371/journal.pone.0066703

**Published:** 2013-06-21

**Authors:** Xuan Wang, Zhongde Mu, Fengqi Shangguan, Ran Liu, Yuepu Pu, Lihong Yin

**Affiliations:** 1 Key Laboratory of Environmental Medicine Engineering, Ministry of Education, School of Public Health, Southeast University, Nanjing, Jiangsu, China; 2 State Key Laboratory of Bioelectronics, School of Biological Science and Medical Engineering, Southeast University, Nanjing, Jiangsu, China; University of Houston, United States of America

## Abstract

A technique was developed for simultaneous detection of fenitrothion (FNT) and chlorpyrifos-methyl (CLT) using a photonic suspension array based on silica colloidal crystal beads (SCCBs). The SCCBs were encoded with the characteristic reflection peak originating from the stop-band of colloidal crystal. This approach avoids the bleaching, fading or potential interference seen when encoding by fluorescence. SCCBs with a nanopatterned surface had increased biomolecule binding capacity and improved stability. Under optimal conditions, the proposed suspension array allowed simultaneous detection of the selected pesticides in the ranges of 0.25 to 1024 ng/mL and 0.40 to 735.37 ng/mL, with the limits of detection (LODs) of 0.25 and 0.40 ng/mL, respectively. The suspension array was specific and had no significant cross-reactivity with other chemicals. The mean recoveries in tests in which samples were spiked with target standards were 82.35% to 109.90% with a standard deviation within 9.93% for CLT and 81.64% to 108.10% with a standard deviation within 8.82% for FNT. The proposed method shows a potentially powerful capability for fast quantitative analysis of pesticide residues.

## Introduction

Organophosphorus pesticides are environmental pollutants in agricultural and non-agricultural products. They have been widely used in agriculture to protect crops against insect damage, as well as in the household to control a number of ecoparasites in domestic animals [1]. In addition, they are also used to protect turf and ornamental plants. There are a few reports in the literature about pollution of drinking water by organophosphorus pesticides [2]. Organophosphorus pesticides are an alternative to organochlorine pesticides but although they degrade more rapidly, they have greater acute toxicity, posing risks to people at high exposure [3]. In recent years, many studies have demonstrated that organophosphorus pesticides are mutagenic, carcinogenic [4]–[7], cytotoxic [8], genotoxic [9], [10], teratogenic [11] and immunotoxic [12]. One of the most important aspects in minimizing the potential hazards of organophosphorus pesticides to humans and the environment is to monitor pesticide residues. The European Union Commission (EU) has set maximum residue limits (MRLs) to control levels of pesticide residues and many countries have established legal directives and monitoring programs to supervise whether or not pesticide residues are compliant with the statutory maximum residue levels. Classical instrumental analytical techniques for pesticide analysis involve gas chromatography [13]–[15], high-performance liquid chromatography [16], gas chromatography coupled with mass spectrometry [17], [18] or liquid chromatography with mass spectrometry [19]. Although chromatography based methods are sensitive and reliable, they require sophisticated equipment, skilled analysts and time-consuming sample preparation steps. Moreover, organic solvents used in the detection process may lead to environmental pollution. Therefore, the development of a rapid, inexpensive, sensitive and high sample throughput analytical method for detection of pesticides is of particular significance and necessity.

As a promising method for selective and sensitive analysis, immunoassays have become indispensable analytical tools in a wide range of applications, including environmental monitoring, clinical diagnosis and food safety [20]. Immunological methods, which are suitable for both laboratory and field analysis, provide a unique opportunity to screen large numbers of samples quickly and effectively. Traditional immunoassays such as enzyme-linked immunosorbent assays (ELISAs) are invariably considered as the gold standard for single analyte measurement. The sensitivity of an ELISA is relatively high, but it has some drawbacks, including numerous washing and preparation steps, large sample volumes, small surface area and long diffusion time required for antigen-antibody binding. Several ELISAs were developed independently for the detection of pesticides [21]–[24]. However, with the demand for multiplexing capability, shorter analysis time, smaller sample volume and higher sensitivity, a number of new techniques are being explored to perform immunoassays [25]. Recently, suspension arrays have increasingly gained attention in multiplex analysis of biomarkers, drug screening, food and environmental monitoring [26]–[32]. Compared with the common single-analyte assays, the multi-analyte suspension array using encoded microbeads as solid supports has the advantages of enhanced detection throughput, shortened analytical time, decreased sampling volume, improved test efficiency, reduced cost and multiplexing capability [33]–[35]. The development of this suspension array technology relies on the design and manufacture of microcarriers, which have both molecular binding abilities and intrinsic identity signatures. In most proposed technologies, the labels are based on fluorescent dyes [36] or quantum dots [37]–[39]. The use of fluorescence dyes limits the number of distinguishable probes and potentially interferes with the signal from the labeling molecules, while quantum dots have drawbacks with respect to biotoxicity and leakage [40].

In our work, silica colloidal crystal beads (SCCBs) were used as supports in a suspension array. These were encoded by the characteristic reflection peak originating from the stop-band of colloid crystal [40]. The code, whose peak position is based on a periodic structure, is very stable and the fluorescent background is low. Additionally, the use of SCCBs greatly improves the sensitivity of the suspension array, because their porous structure provides a higher surface-to-volume, which can further enhance the extent of reactions.

In this paper, we report on a photonic suspension array based on SCCBs for multiplex detection of organophosphorus pesticides (using FNT and CLT as model analytes). The selected pesticides have become important pesticides for controlling insects and acarids in many agricultural crops in China because most highly toxic and high-residue organophosphate pesticides, for example, methamidophos, parathion, and methyl parathion, were banned for use on crops by the Chinese government. FNT [O,O-dimethyl O-(3-methyl-4-nitrophenyl)-phosphorothioate], as a contact insecticide and selective acaricide, is a contact-acting organophosphorus pesticide that inhibits acetyl cholinesterase activity, thus disrupting the nervous system [41]. It is widely used against insect pests and mites on cereals, cotton, orchard fruits, rice, vegetables and forests [42]. CLT [O,O-dimethyl-O-(3,5,6-trichloro-2-pyri-dyl) phosphorthioate] is a broad-spectrum organophosphorus pesticide that is used to control pests in grain storage, water and a variety of leafy crops [43]. Because the selected pesticides are toxic and extensively applied, they are classified as restricted pesticides with a strict maximum residue limited standard for use in many countries. The SCCBs with different reflection peak positions were then used as microcarriers for corresponding anti-FNT and anti-CLT monoclonal antibodies and their encoding was characteristic reflection peak originating from the stop-band of the colloidal crystal. The photonic suspension array for simultaneous detection of the selected pesticides was a competitive immunoassay based on the increase of the fluorescein signals from the conjugates of pesticides (FNT-OVA and CLT-OVA) labeled by fluorescein isothiocyanate (FITC) when they were bound by the specific monoclonal antibodies of pesticides immobilized on different types of SCCBs (Fig. S3 in the Supporting information). If the sample contained the target pesticides, the FITC-labeled conjugates would compete with the target pesticides for binding with the antibodies and the fluorescein signals would decrease when more target pesticides were available. The proposed method based on SCCBs was accurate and displayed the advantages of simplicity, rapidity, sensitivity and low-cost.

## Materials and Methods

### Materials

CLT standard, FNT standard and bovine serum albumin (BSA) were brought from Sigma Chemicals (USA). 3-glycidoxypropyltrimethoxysilane (GPTMS) was purchased from Alfa Aesar Co. Mouse monoclonal anti-FNT antibody, mouse monoclonal anti-CLT antibody, FITC labeled FNT-OVA (FF competitor) and FITC labeled CLT-OVA (FC competitor) were obtained from Zoonbio Biotechnology Co. Ltd (Nan Jing, China). All other reagents were of the best grade available and used as received.

All buffers were prepared with water purified in a Milli-Q system (Millipore, Bedford, MA). Washing buffer (PBST) was PBS (0.01 M, pH 7.4) containing 0.05% v/v Tween 20. Blocking buffer was PBS containing 5% (v/v) bovine serum albumin.

### Instrumentation

Photographs of SCCBs were taken with an optical microscope (OLYMPUS BX51) equipped with a CCD camera (Media Cybernetics Evolution MP 5.0). The microstructures of SCCBs were characterized by scanning electron microscopy (SEM, Hitachi, S-300N). Antigen-antibody reaction was carried out in the constant temperature shaker (Eppendorf Thermomixer comfort 5355). Reflection spectra of SCCBs were recorded by a microscope equipped with a fiber optic spectrometer (Ocean Optics, USB2000). Fluorescence spectra of SCCBs were recorded by a microscope equipped with a fiber optic spectrometer (Ocean Optics, QE65000).

### Fabrication of SCCBs

Monodisperse silica nanoparticles were synthesized by the Stöber method [44]. The microfluidic device used to generate SCCBs was custom made [45]. Two types of SCCBs with different characteristic reflection spectra were produced using an improved capillary microfluidic device, which is more flexible, has lower probability of channel congestion and is easier to control when fabricating SCCBs with different sizes [46]. Fig. S1 (Supporting information) shows images of the two types of SCCBs.

### Probes Immobilization

The SCCBs were coated with mouse monoclonal antibody using a covalent bonding method [47]. First, SCCBs were treated with piranha solution (30% hydrogen peroxide and 70% sulfuric acid) for 48 h to increase the number of silica hydroxyls on the SCCBs. The SCCBs were then incubated with a toluene solution of GPTMS (10%) for 12 h to modify the surface with epoxy groups. After the coupling reaction, the modified SCCBs were rinsed, in turn, with toluene, ethanol and water. Subsequently, as shown in Fig. S2 (Supporting information), exposed active epoxy groups were formed on the surface of SCCBs and further reacted with mouse monoclonal antibodies in PBS at 4°C for 12 h. After washing with PBST, the unreacted epoxy groups on the surface of SCCBs were blocked with 5% (v/v) BSA in PBS. For multiplexed immunoassays, two types of SCCBs with different reflection peak positions, were modified with anti-CLT antibody and anti-FNT antibody, respectively.

### Multi-analyte Assay

The proposed method was based on a competitive immunoassay, which was performed as follows. Two types of modified SCCBs were put into one test tube and incubated with a mixed solution containing CLT, FNT, FC competitors and FF competitors for 30 min. During the incubation process, the test tubes were shaken at 37°C. In this period, competitions were carried out between the pesticides and competitors in solutions (CLT and FC competitors, FNT and FF competitors) for binding to a fixed amount of mouse monoclonal antibodies on SCCBs. When increasing concentrations of pesticides, the fluorescence intensities reduced because pesticides inhibited the antibodies binding to the competitors. Because the test tube was flat-bottomed, SCCBs could roll at the bottom of the tube, ensuring that antibodies on SCCBs were in full contact with the targets. After washing with PBST, fluorescence intensities of SCCBs were measured.

### Specificity of the Photonic Suspension Array

The specificity of the suspension array was assayed by its exposition to various chemicals, namely: chloryrifos, bromophos, 3,5,6-trichloro-2-pyridinol, triazophos, methidathion, fenthion, paraoxon, chlorthion, parathion and parathion-methyl at concentrations of 1024 ng/mL. The concentrations of the chemicals were calculated using standard curves and the cross-reaction rates were calculated by the concentration values divided by 1024 ng/mL.

### Accuracy (Analysis of Spiked Samples)

Grape, lettuce and cabbage from a local market and tap water from our laboratory were chosen for recovery studies. CLT and FNT standard stock solutions were prepared at 10, 100 and 1000 ng/mL. Grape peel, lettuce and cabbage leaves were chopped into fine pieces and 1 mL of each solution was added to 1 g of the samples. After standing overnight at 4°C for 24 h, the samples were shaken in 5 mL of methanol for 1 h and then filtered through a filter paper. Methanol was evaporated to dryness and the residue was extracted with 10 mL of 10% methanol-PBS (The final concentrations of pesticides were 1, 10 and 100 ng/mL). The extract was then analyzed using our suspension array.

## Results and Discussion

### Characterization of the Photonic Suspension Array

**Figure 1:**
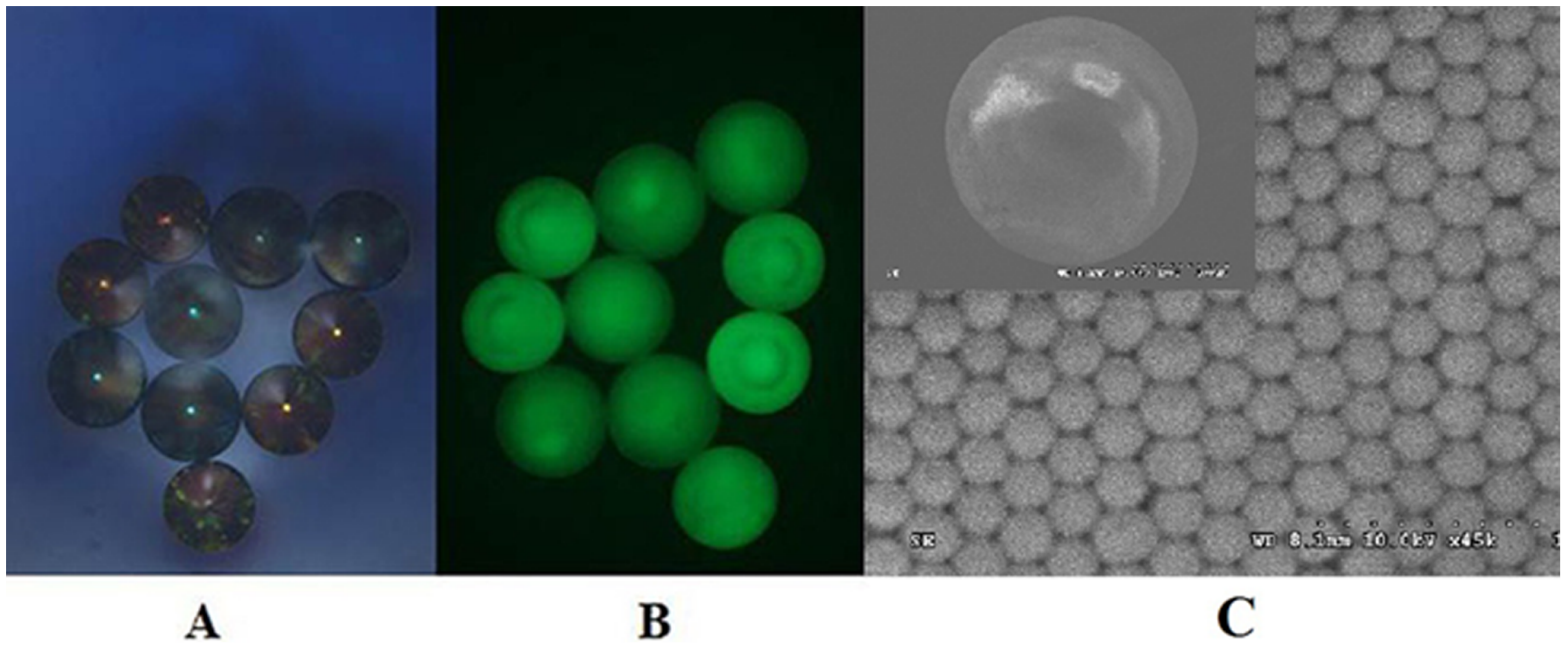
SEM images of SCCBs and images under a bright field and a dark field. SEM images of SCCBs (C) and images of multiplex detection obtained under a bright field (A) and dark field (B). Blue: FNT, yellow: CLT.

A photonic suspension array based on SCCBs was developed for simultaneous detection of the selected pesticides using antibodies covalently immobilized on the SCCBs surface. Fig. 1C shows SEM images of the SCCBs. The SCCBs offer dual advantages in this application. First, the SCCBs, which were derived via the assembly of monodisperse colloidal nanoparticles in droplet templates, were encoded with the characteristic reflection peak originating from the stop-band of the colloidal crystal. Because the peak position depends on the periodic structure, such encoding is very stable. Second, the increased binding capacity of the porous structure of the SCCB microcarriers improved the orientation of the immobilized antibodies and lowered mass transfer resistance. The immobilized antibodies were distributed uniformly within the particles, which facilitated the approach of immunoreagents and improved the kinetics of the antibody-antigen interaction. This improved experimental layout allowed us to obtain a high sensitivity SCCB suspension array.

To apply the photonic suspension array, a simple platform was developed by incorporating a fiber optic spectrometer into a microscope for decoding and detection of the SCCBs (Fig. S4 in the Supporting information). When the SCCBs were exposed to white light under normal incidence through the microscope, the reflection peaks could be detected and the peak positions recorded for decoding. The fluorescence signals could be measured by replacing the input white light with blue light at a wavelength of 488 nm. Fig. 1A and Fig. 1B show images of multiplex detection obtained under bright and dark fields, respectively.

**Figure 2:**
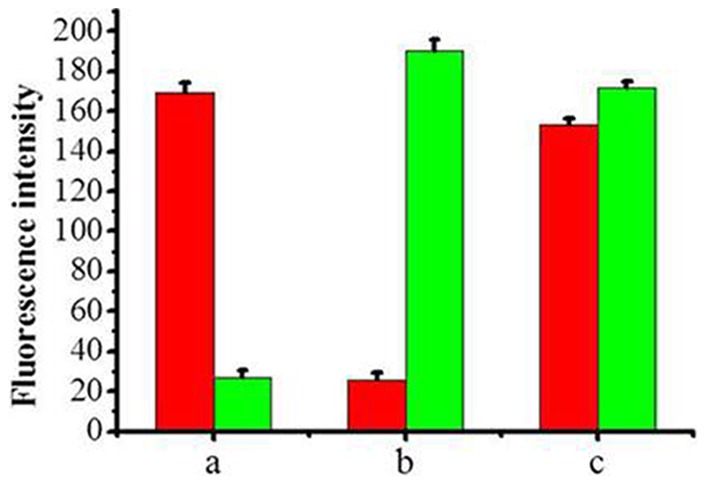
Response of the suspension array system to both competitors. Fluorescence intensity of the two types of SCCB modified by anti-FNT antibody and anti-CLT antibody, respectively, for FF competitors (a) or FC competitors (b) or a mixture of FF and FC competitors; the red and green bars represent the response of the system to FF and FC competitors, respectively.

Prior to multiple analysis, it was essential to investigate the cross-reactivity between the two immobilized antibodies and both FF competitors and FC competitors. When each type of modified SCCBs was incubated with a single competitor (FF competitors or FC competitors), the suspension array system showed only a single response, corresponding to its competitor, with no response for the other competitor (Fig. 2a and b), indicating negligible cross-reactivity. When incubated with both FF competitors and FC competitors, our suspension array showed responses to both, indicating that the two antibodies were successfully immobilized onto the surface of SCCBs (Fig. 2c). Hence, the suspension array system could be used to detect the selected pesticides, simultaneously.

### Optimization of Competitive Fluorescence Immunoassay

**Figure 3:**
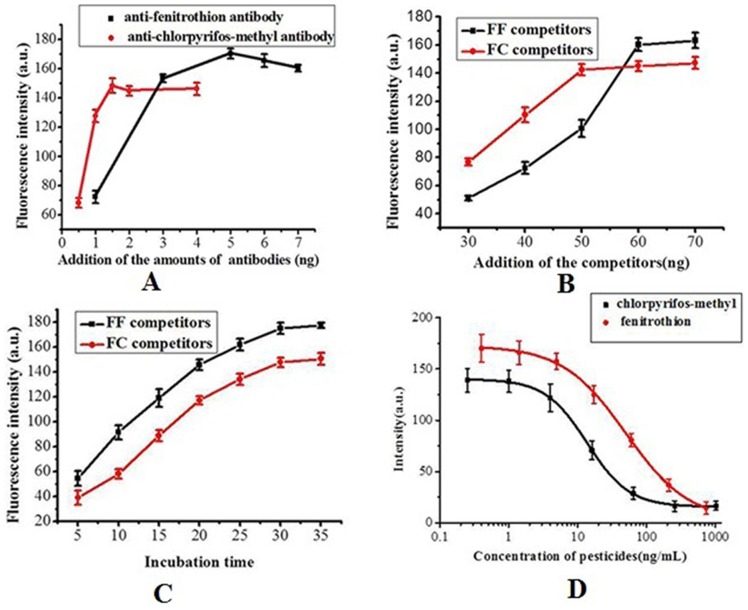
Optimization of experimental conditions and standard curves. Effects of different amounts of mouse monoclonal antibodies (A) and competitors (B) on fluorescence intensities, effects of incubation time on fluorescence intensities (C) and standard curves of the photonic suspension array (D). Each point was obtained by detecting 5 SCCBs.

The amounts of antibodies and competitors added and the incubation time affected the sensitivity of our suspension array. In Fig. 3A, differences are shown between the fluorescence intensities and the amount of antibodies added. With increasing addition of antibodies, the fluorescence intensities increased and showed clear points of inflection. Hence, optimal amounts of the two antibodies were chosen as 1.50 and 5 ng in subsequent experiments.

To select the optimal amounts of FF competitors and FC competitors, SCCBs were incubated in mixed solutions containing variable amounts of competitors. As shown in Fig. 3B, the fluorescence intensities increased with increasing amounts of FF and FC competitors and tended to plateau at 60 and 50 ng, respectively, indicating that these amounts of competitor effectively blocked all of the antibodies affinity sites in each case. Therefore, 60 ng of FF competitors and 50 ng of FC competitors were used in subsequent experiments.

The incubation time is important for maximizing the speed of an immunoassay. With increasing incubation time, both fluorescence intensities increased and approached their maximum values after an incubation time of 30 min. This time was therefore chosen for the competitive immunoassay (Fig. 3C). Compared with the 1∼3 h at 37°C required for a traditional microwell plate ELISA, our suspension array required a shorter incubation. The large specific interfacial area and short diffusion distance of the suspension array enabled the immunoreagents to easily access the bound antibodies and increased the rate of immunoreaction.

### Standard Dose-response Curve

Standard curves for our suspension array were measured at known concentrations of FNT and CLT standard solution under the optimal conditions identified above. As indicated in Table 1 and Fig. 3D, both coefficients of determination (R^2^) for the standard curves were more than 0.99, which indicates a good correlation between fluorescence intensities and the logarithms of FNT and CLT concentration. The detection ranges of the selected pesiticides were 0.25 to 1024 ng/mL for FNT and 0.40 to 735.37 ng/mL for CLT. Because there were no significant differences between the obtained fluorescence intensities of the blank control and the groups of the minimum detectable concentration (Min DC), the Min DC could be considered as the LOD. Thus, the LODs of FNT and CLT were 0.25 and 0.40 ng/mL, respectively, which were much lower than MRLs (10 ng/mL for FNT and 50 ng/mL for CLT) regulated by the European Union in fruits and vegetables [48]. The method developed in this study might achieve the detection requirement for detecting the selected pesticides in real food samples. Compared with other methods (Table 2), the LODs obtained for the selected pesticides using the suspension array were lower than those reported determination methods in the literatures [23], [49]–[56]. This indicated that the proposed method held promising applications in environmental and food monitoring.

**Table 1:** Standard curves for simultaneous detection of FNT and CLT and fluorescence intensities for the blank control and the LOD.

Pesticide	Standard curve	R^2^	Intensity ( 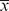 ± SD, n = 5)	
			Blank control	Min DC
FNT	y = 29.820+104.482/[1+(x/51.209)^0.622^]	0.998	165.24±3.35	159.12±2.45
CLT	y = 79.109+81.055/[1+(x/33.997)^0.766^]	0.997	139.87±3.53	132.11±3.22

**Table 2:** Comparison with methods reported in the literature.

Analyte	LOD (ng/mL)	Method	Reference
FNT	1.60	ELISA	[23]
CLT	0.32	ELISA	[48]
FNT	2.68	LC–MS/MS	[56]
CLT	1.60	LC–MS/MS	[56]
FNT	0.50	spectrophotometry	[50]
CLT	132.91	immunochromatographic assay	[51]
FNT	1.40	HPLC/UV	[52]
CLT	2.52	GC/NPD	[53]
FNT	50	HPLC/UV	[54]
CLT	2.0	GC/NPD	[55]
FNT	0.25	suspension array	In this paper
CLT	0.40	suspension array	In this paper

### Evaluation of Cross Reactivity

Considering specificity and reliability of the immunoassay, cross-reaction is a critical analytical parameter. The chemicals chosen to estimate the specificity of the suspension array were chloryrifos, bromophos, 3,5,6-trichloro-2-pyridinol, triazophos, methidathion, fenthion, paraoxon, chlorthion, parathion and parathion-methyl. To examine the cross-reactivity between the two antibodies and their non-related analytes, solutions of these other chemicals with concentrations of 1024 ng/mL were analyzed by the suspension array. For anti-CLT antibody, CLT was considered as the specific analyte whilst the other reagents were classified as cross-reactants, and similarly for the anti-FNT antibody. Table S1 (Supporting information) shows the cross-reaction rates of the chemicals with the two antibodies, showing that the cross-reactivity values of the various potential cross-reactants were very small and fell below 5%. Thus, it could be considered that the suspension array is very specific.

### Reproducibility and Stability of the Immunoassay Based on SCCBs

To investigate the reproducibility of the photonic suspension array, we repeatedly assayed ten times at two different concentrations of the selected pesticides. The relative standard deviations (RSDs) were 9.12% and 6.50% for 100 and 400 ng/mL CLT, 7.83% and 5.16% for 50 and 200 ng/mL FNT, showing acceptable reproducibility.

When the photonic suspension array was not in use, SCCBs were stored in PBS (pH 7.4) at 4°C. No obvious change was observed after storage for at least one year for SCCBs without probe immobilization and for at least one week for SCCBs with immobilized probe. Hence, combined with its multiplex analysis capability, the photonic suspension array here presented is a very promising analytical assay for several fields of application.

### Accuracy (Analysis of Spiked Samples)

Spiked samples were used to measure the accuracy of the assay. The total number of samples assayed was sixteen (n = 3). Samples were extracted with methanol, followed by evaporation of the solvent and then dissolution of the residue in 10 mL of 10% methanol-PBS. As shown in Table 3, recoveries of 82.35% to 109.90% for CLT and 81.64% to 108.10% for FNT were obtained at all levels with RSDs of 3.22% to 9.93% and 2.93% to 8.82%, respectively. In the pesticide analysis field, recovery rates in the range of 70∼120% are considered to be acceptable and can be extended to routine analysis, as recommended by the EU Commission guideline about determination of pesticide residues in food [57]. Therefore, our suspension array was sufficiently accurate and could be suitable for the quantification of the selected pesticides in fruits, vegetables and water.

**Table 3:** Recovery test of CLT and FNT in real samples.

Samples	Level spiked(ng/mL)	Average recovery ±SD(%)	
		CLT	FNT
	0	–	–
	1	86.65±5.25	89.23±4.38
Grape	10	82.35±3.97	81.64±5.93
	100	89.62±6.32	86.83±7.58
	0	–	–
	1	97.32±7.68	82.27±6.74
Lettuce	10	93.23±6.43	86.77±3.45
	100	86.43±8.37	90.54±5.34
	0	–	–
	1	88.37±6.54	92.18±3.39
Cabbage	10	94.33±7.86	85.11±2.93
	100	82.75±3.22	83.83±5.44
	0	–	–
	1	108.52±7.79	102.35±3.63
Water	10	109.90±5.70	105.90±6.74
	100	104.4±9.93	108.10±8.82

“–”Refers to the undetectable concentrations or no results.

### Conclusions

We successfully developed a simple photonic suspension array for simultaneous detection of the selected pesticides. The SCCBs were proven to be effective microcarriers with a stable encoding strategy for developing high-throughput multiplex assays. The high sensitivity obtained, when compared with other techniques, was due to the enhanced binding capacity of the porous SCCB structure. The suspension array has acceptable stability, reproducibility and accuracy. The high sensitivity and specificity of the proposed system fully satisfies the requirements of analytical fields. The photonic suspension assay provides significant advantages over single analyte tests in terms of cost, labor, test throughput and convenience but the most significant advantage of this method is its capability for multiplex analysis. The photonic suspension array is very promising not only for the detection of macromolecules (proteins and nucleic acids) but provides a novel pathway for analysis of small molecules such as pesticides, veterinary drugs and toxicants.

## Supporting Information

Figure S1 **Photographs of two kinds of SCCBs and their reflection spectra with reflection peaks at 505 and 575 nm, respectively.**
(TIF)

Figure S2 **Scheme for immobilization of antibodies on the surface of SCCBs.**
(TIF)

Figure S3 **Scheme for the SCCBs based photonic suspension array. (1) Incubation for competitive binding (30 min). (2) Washing and detection.**
(TIF)

Figure S4 **The platform was used for decoding and detection of the SCCBs.**
(TIF)

Table S1 **Cross-reactivity of chemicals determined by the photonic suspension array.**
(DOCX)
