# Supplementary figures and images for: Simultaneous Detection of Fenitrothion and Chlorpyrifos-Methyl with a Photonic Suspension Array
Source: PLoS One. 2013 Jun 21;8(6):e66703. doi: 10.1371/journal.pone.0066703 (PMC3689689; doi:10.1371/journal.pone.0066703)

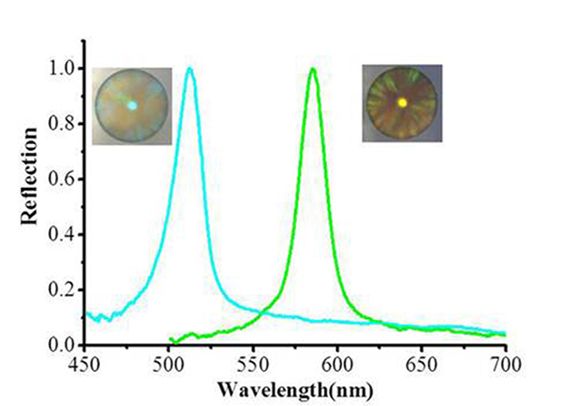

Supplement: Figure S1 — Photographs of two kinds of SCCBs and their reflection spectra with reflection peaks at 505 and 575 nm, respectively. (TIF) [file pone.0066703.s001.tif]

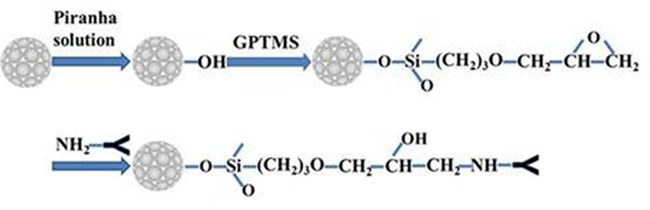

Supplement: Figure S2 — Scheme for immobilization of antibodies on the surface of SCCBs. (TIF) [file pone.0066703.s002.tif]

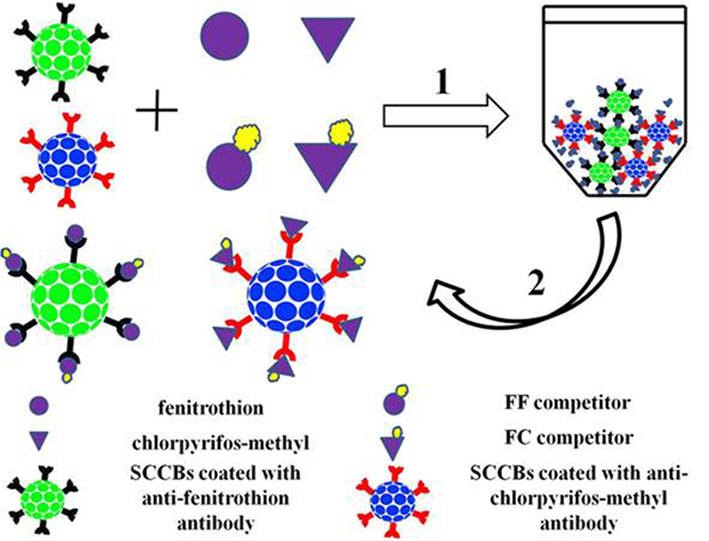

Supplement: Figure S3 — Scheme for the SCCBs based photonic suspension array. (1) Incubation for competitive binding (30 min). (2) Washing and detection. (TIF) [file pone.0066703.s003.tif]

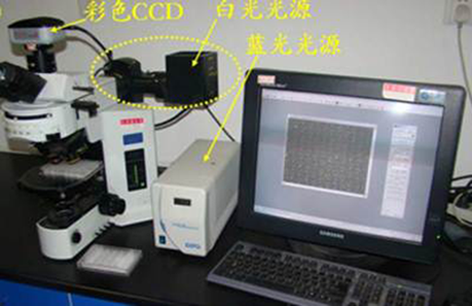

Supplement: Figure S4 — The platform was used for decoding and detection of the SCCBs. (TIF) [file pone.0066703.s004.tif]
