# Supplementary material for: Simultaneous Detection of Fenitrothion and Chlorpyrifos-Methyl with a Photonic Suspension Array
Source: PLoS One. 2013 Jun 21;8(6):e66703. doi: 10.1371/journal.pone.0066703 (PMC3689689; doi:10.1371/journal.pone.0066703)
Supplement: Table S1 — Cross-reactivity of chemicals determined by the photonic suspension array. (DOCX) [file pone.0066703.s005.docx]

Table S1. Cross-reactivity of chemicals determined by the photonic suspension array

| Chemicals |  | Cross-reactivity (%) | |
| --- | --- | --- | --- |
|  | Structures | 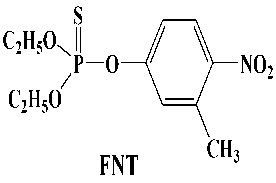 | 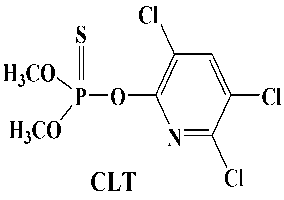 |
| Chloryrifos | 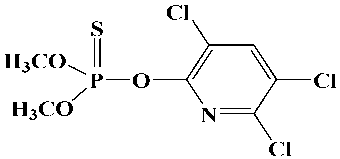 | <0.10 | 4.96 |
| Bromophos | 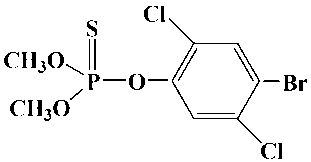 | <0.10 | 2.54 |
| 3,5,6-Trichloro-2-pyridinol | 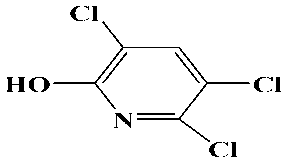 | <0.10 | <0.10 |
| Triazophos | 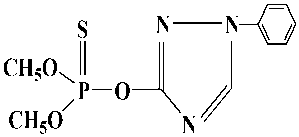 | <0.10 | <0.10 |
| Methidathion | 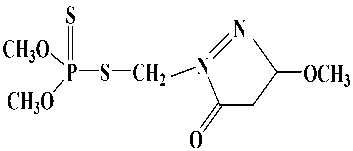 | <0.10 | <0.10 |
| Fenthion | 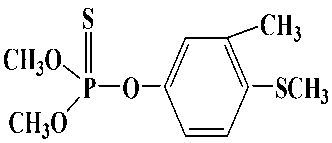 | 4.72 | <0.10 |
| Paraoxon | 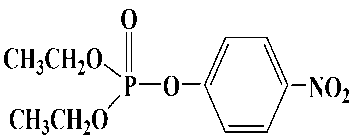 | 2.11 | <0.10 |
| Chlorthion | 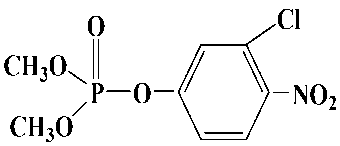 | <0.10 | 0.52 |
| Parathion | 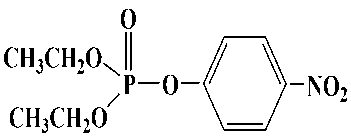 | 1.25 | 0.64 |
| Parathion-methyl | 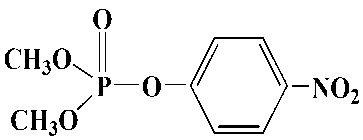 | 2.49 | 0.95 |
